# Damage Monitoring of Structural Resins Loaded with Carbon Fillers: Experimental and Theoretical Study

**DOI:** 10.3390/nano10030434

**Published:** 2020-02-29

**Authors:** Giovanni Spinelli, Patrizia Lamberti, Vincenzo Tucci, Liberata Guadagno, Luigi Vertuccio

**Affiliations:** 1 Department of Information and Electrical Engineering and Applied Mathematics, University of Salerno, Via Giovanni Paolo II, 84084 Fisciano (SA), Italy; gspinelli@unisa.it (G.S.); plamberti@unisa.it (P.L.); vtucci@unisa.it (V.T.); 2 Department of Industrial Engineering, University of Salerno, Via Giovanni Paolo II, 84084 Fisciano (SA), Italy; lguadagno@unisa.it

**Keywords:** carbon nanoparticles, damage detection, structural health monitoring, electrical properties

## Abstract

In the present study, nanocomposite materials for structural applications with self-sensing properties are proposed. In particular, suitable processing of epoxy resins filled with carbon nanotubes and expanded graphite characterized by very different aspect ratio leads to nanocomposite systems with high glass transition temperatures and remarkable values of the gauge factor. In particular, this notable property ranges between four, for composites filled with one-dimensional nanofiller, and 39 for composites with two-dimensional (2D) graphite derivatives. The greater sensitivity of the 2D system against permanent deformations is interpreted on the basis of an empirical mathematical model and morphological descriptions. The larger inter-contact area among the graphite layers determines a larger contact resistance change than that occurring among carbon nanotubes. The proposed systems turn out to be very advantageous in strain-sensor applications where damage detection is a key requirement to guarantee the reliability of the structures and the safety of the end-users.

## 1. Introduction

The health monitoring of structural parts is particularly relevant in the transport sectors and in particular in the aeronautic and automotive ones. In these fields, damage detection is a safety issue that is addressed through preventive and/or corrective maintenance actions. The structural health monitoring (SHM) processes involve high direct and indirect costs associated with the implementation of suitable characterization strategies for the structures. For example, as it concerns the aeronautic sector, the stalling of the aircraft to carry out inspections on structures leads to economic losses due to the reduction in flight hours. Therefore, in this context, SHM becomes an issue that should be economically viable. In the aircraft industry, at present, monitoring techniques are based on pessimistic predictions, periodic inspections and non-destructive testing (NTD). NDT aircraft inspections are generally performed manually, although most of them (visual inspection, ultrasonic inspection, eddy current, acoustic emission radiography, thermography, and shearography) are long-established approaches [1]. All these currently used techniques require the integration of appropriate systems inside the structure, generally different types of actuator-sensors. By using analytical procedures applied to mechanical tests, these systems permit an estimate of the damage caused by fatigue cycles.

Smart structures and materials pave the way towards new valid strategies for damage monitoring. With the recent advent of nanotechnology science, carbon nanofillers have been used for the development of multifunctional nanocomposites with integrated self-sensing properties. Based on these nanocomposites, SHM devices able to detect damage in structural materials can be produced [2,3,4,5,6,7,8]. More specifically, the inclusion of electrically conductive nanofillers in epoxy resins characterized by mechanical performances suitable for fulfilling structural requirements confers them piezoresistive properties. The variation of the residual electrical resistances of the nanocomposites allows identifying in a reliable manner permanent damages due to mechanical stresses [4,9]. More recently, 2D carbon fillers have been taken into consideration in addition to the well-known 1D carbon nanotubes (CNTs) for the production of nanocomposites with piezoresistive properties. Graphene-based nanoparticles, graphite and its derivatives (expanded and exfoliated graphites) incorporated in polymeric matrices lead to the development of smart materials that find significant practical applications in various fields, such as the aeronautical industry [10,11], flexible electronics [12], radiation protection [13], electromagnetic interference (EMI) or electromagnetic compatibility (EMC) [14], self-heating systems [3] and for strain sensing in structural health monitoring (SHM) systems [15]. This last application is widely discussed in the literature [6,7,8,16]. In this context, a great variety of materials have been investigated. Nevertheless, the complete understanding of the piezoresistive properties is not yet fully understood, since such properties are affected by several factors, such as, for example, the concentration and dispersion of 2D fillers, the manufacturing methods, the nanofiller functionalizations, the inter-sheet joining and orientation of platelets in the final manufactured composite [10,17,18,19]. Furthermore, from an industrial point of view, targeted usages of these systems cannot be adequately addressed without reference to the nature and characteristics of the materials, which must satisfy specific requirements. For instance, in the aircraft manufacturing sector, mechanical requirements and operating conditions are very stringent. For primary structures, the usable materials are limited to a particular class of epoxy resins characterized by glass transition temperatures higher than 180 °C, suitable values of the storage modulus in the range of service temperature, resistance to fatigue cycles etc. In light of these very severe constraints, the present work proposes a suitable and viable solution. In particular, two fundamental aspects of the above-mentioned criticalities are faced. Firstly, the development and selection of the best self-sensing system is able to preserve the mechanical performance of the matrix. Secondly, the theoretical interpretation of the results is proposed not only to provide an in-depth knowledge of the involved parameters, but also to make effective predictions on the efficacy of self-sensing response in terms of sensitivity. Remarkable performances in terms of the piezoresistive response of composites including 1D and 2D nanofillers are achieved. An empirical mathematical model is adopted as a valid support for the interpretation of the electro-mechanical mechanisms responsible of the obtained results concerning the piezo-resistive response. The paper is organized as follows. In Section 2 the description of the adopted materials and production procedures is provided; in Section 3, the obtained experimental results are presented, together with their interpretation based on a morphological study and model predictions. In Section 4, the conclusions and possible developments are proposed.

## 2. Materials and Methods

In the compounds used in this study, the precursor was based on the “3,4-Epoxycyclohexylmethyl-3’,4’-epoxycyclohexane carboxylate” (ECC), whereas the hardener agent was based on the “Methyl hexahydrophthalic anhydride” (MHHPA). Gurit (Gurit Holding Wattwil, Svizzera) supplied these components. The carbon nanotubes (GRAPHISTRENGTH C100) were obtained from ARKEMA (ARKEMA Colombes, France). The carbon purity was >90% with a weight loss at 105 °C <1% (value obtained by thermogravimetric analysis (TGA)). Multi-walled carbon nanotubes (MWCNTs) are characterized by an outer mean diameter ranging from 10 to 15 nm, a length ranging from 0.1 to 10 μm and a number of walls varying between 5 and 15. The expanded graphite (EG) was obtained from Superior Graphite (Superior Graphite Co., Chicago, IL, USA). The expanded graphite is characterized by the ash of 0.05%, a true density of 2.25 g/m^2^ and a surface area (BET) of 18.2 m^2^/g.

In order to achieve a uniform dispersion, the filler was embedded into the precursor by using ultra-sonication for 20 min (Hielscher model UP200S-24 kHz high power ultrasonic probe, Hielscher Ultrasonics, Teltow, Germany). An amount of hardener was added to filler/precursor mixture with a ratio by weight precursor/hardener of 1:1. The obtained mixture was mixed by magnetic stirring for 20 min at room temperature and subsequently degassed for 2 h at room temperature. The mixtures were obtained at different percentages for both fillers (MWCNT and Expanded Graphite). The obtained mixtures were cured adopting the following curing cycle: 1 h at 80 °C + 20 min at 120 °C + 1 h at 180 °C. The dynamic mechanical thermo-analyzer (Tritec 2000 DMA-Triton Technology, Grantham, UK) was adopted for the investigation of dynamic mechanical properties according to the specifications summarized in Table 1.

Morphological analysis regarding the dispersion of fillers within the matrix was investigated by means of scanning electron microscopy (SEM) apparatus (JSM-6700F, JEOL, Aksishima, Japan) on etched samples obtained with a procedure reported in Vertuccio et al. [20]. The electro-mechanical tests were carried out on filled formulations at 0.5% and 2.0% by weight of carbon nanotubes and on formulations at 2% by weight of expanded graphite.

The samples to be characterized experimentally were produced following a method already described in Ref. [4] and in accordance with the specifications of ASTM D638 standards [21] (American Society for Testing and Materials, ASTM) for mechanical tests performed by using a Dual Column Tabletop Testing Systems (INSTRON, series 5967-INSTRON, Norwood, MA, USA) configured with a crosshead speed of 1 mm/min for both loading and unloading. The resultant force was converted to axial stress (σ), while mechanical strain (ε) was determined as the machine crosshead displacement normalized by the gage length of the specimen under test. In order to exclude slipping phenomena, the local elongation was detected by means of a commercial strain gauge (RS 632-180, RS PRO, Corby, UK) conveniently stuck to one side of the specimen. Its gauge resistance changes were monitored with a Multimeter 3458A (Agilent, Santa Clara, CA, USA). The current-voltage (I-V) characteristics were measured with a two-probe method on electrical contacts realized on the sample surface with silver paint (RS 196-3600, RS PRO, Corby, UK) using an electrometer Keithley 6517A (Keithley Instruments, Cleveland, OH, USA) that acted as power supply and ammeter. Such a measurement method was considered suitable in the literature for the electro-mechanical characterization [22,23]. The contact resistance can be ignored since lower than the overall electrical resistance of the specimens (several kΩ). A preliminary DC electrical characterization of all formulated nanocomposites was carried out by evaluating conductivity and percolation threshold on specimens with a round shape (2 mm and 50 mm of thickness and diameter, respectively). At least 3 samples were tested for each composition.

## 3. Results and Discussion

### 3.1. Electrical and Mechanical Properties

The percolation theory can be applied to explain the electrical conducting behavior of composites obtained by including conductive fillers in insulating matrices. The composite undergoes an insulator-to-conductor transition when the conductive filler loading is increased up to the achievement of a critical concentration, the so-called electrical percolation threshold (i.e., EPT). Starting from this filler amount, the electrical conductivity of the composite exhibits a sharp increase of several orders of magnitude with respect to the value exhibited by the pure matrix. This effect is due to the formation of continuous conductive paths that allow the electron flow through the material. Below the percolation threshold, the electrical network is not yet established and the overall electrical performance is that of the host matrix with conductivity values of few pS/m, as expected for insulating material. 

**Figure 1:**
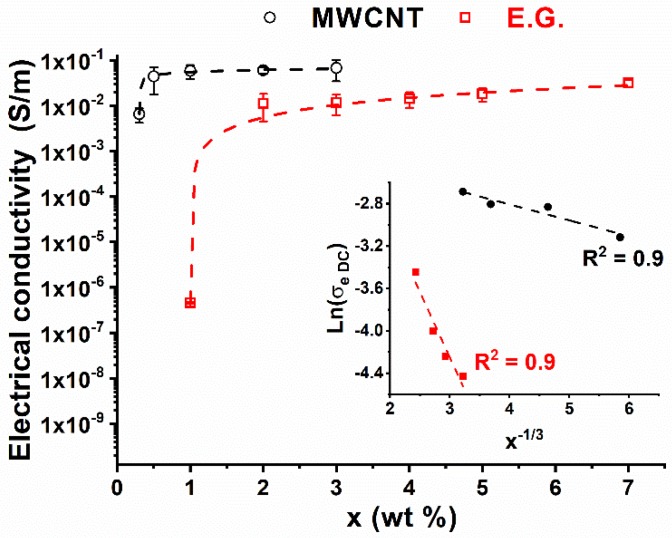
DC volume conductivity of the nanocomposites versus filler weight percentage.

Figure 1 shows the electrical conductivity (σ_e_) as a function of the filler concentration (wt.%) (x) for both nanocomposites based on MWCNTs and EG. 

For concentrations above the EPT, the conductivity varies with the filler loading (i.e., x) in agreement with a scaling law of the type:

(1) $$\sigma_{e}=\sigma_{e0}{\left(x-x_{c}\right)}^{t}$$

where σ_e0_ is the intrinsic filler conductivity, x_c_ is the weight amount of the filler corresponding to the percolation threshold and t is an exponent depending on the system dimensionality, i.e., the structural organization of the filler inside the host matrix [24,25].

The two systems differ in both the electrical conductivity values and the electric percolation thresholds. EPT values are about 0.3 and 1.0 wt.% for nanocomposites obtained with MWCNTs and EG, respectively. Following a classical approach widely discussed in the literature [26,27,28] for confirming that the tunneling effect is the main electrical transport mechanism in nanofilled polymers, the inset of Figure 1 reports the electrical conductivity (in natural logarithmic scale) against x^−1/3^ for concentrations (x) above the EPT for both carbon-based systems. The dashed lines are linear fitting of the experimental data (red and black markers). Due to the occurrence of the linearity condition (R^2^ close to 1), i.e., Ln (σ_eDC_) ∝ x^−1/3^, it is reasonable to sustain that the electrical conduction in the analyzed composites is mainly governed by an electron tunneling mechanism between the clusters of CNTs. In order to get significant conductivity values (of the order of mS or more), it has been shown that the average distance between neighbor CNTs has to be lower than 2 nm [29]. The system with MWCNTs is more conductive than that filled with the 2D systems of about one order of magnitude at the filler loadings chosen for electro-mechanical tests (i.e., 2 wt.%). Comparable values for the electrical conductivity (3.4 × 10^−2^ S/m and 3.2 × 10^−2^ S/m) are observed at the maximum concentration used that is 3 wt.% for the MWCNT-composite and 7 wt.% for the EG system. The piezo-resistive behavior of the composites is closely related to the electrical resistance of the sample; the higher the electrical resistance, the higher the sensitivity of the system to strain variation induced by an applied load. One way to quantify the sensitivity of the system to strain variations is to evaluate the Gauge Factor (GF) defined as:

(2) $$\mathrm{GF}=\frac{\Delta R}{R_{0}}\times \frac{1}{\varepsilon }$$

where ΔR = R−R_0_ is the resistance variation with respect to the value in resting-state R_0_ (no-load applied) and ε is the measured strain. The GF of the composites loaded with carbon filler, as reported in different works [30,31,32], decreases with increasing the carbon filler percentage. In more detail, when the amount of the filler is low, close to the EPT, a higher value of the GF is obtained [9]. In this work, the piezoresistive behavior was analyzed for systems having concentrations around the percolation threshold, i.e., 0.5% for the 1D MWCNT system and 2% for the 2D composite. Furthermore, a further set of samples based on MWCNTs at a concentration of 2% was produced in order to compare the 1D and 2D systems with the same concentration. 

**Figure 2:**
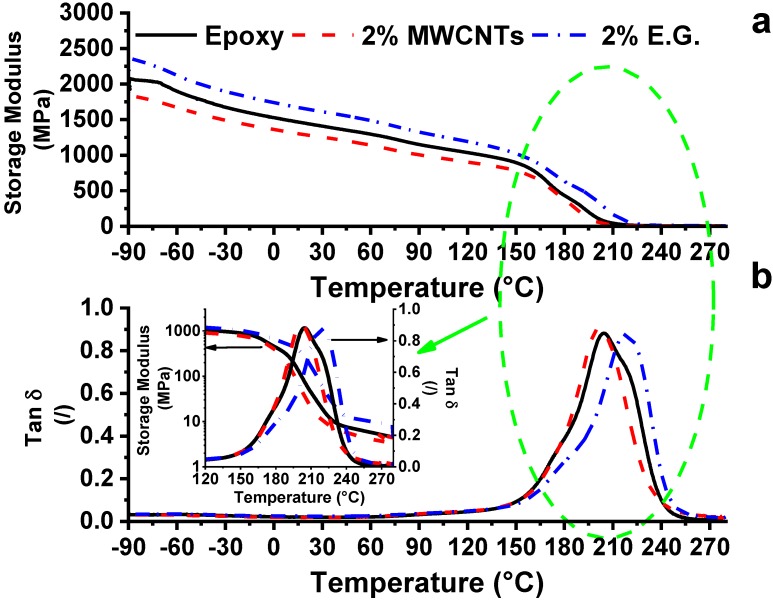
Storage Modulus (**a**) and the loss factor (tan δ) (**b**) of the unfilled epoxy formulation and epoxy formulations at a loading of 2% by weight of multi-walled carbon nanotubes (MWCNTs) and expanded graphite (EG) composites.

Figure 2 shows dynamic mechanical analysis for systems with and without fillers: (a) storage modulus and (b) loss factor (tan δ) vs. temperature for neat resin (i.e., epoxy) and nanocomposites filled with 2.0 wt.% of MWCNTs and expanded graphite, respectively.

For all systems, the glass transition temperature ranges between 180 and 240 °C, thus confirming their reliability as structural materials for aeronautical components. The trends of the curves in Figure 2a show a progressive decrease in the modulus of up to 160 °C; after that, a more marked drop occurs, due to the glass transition temperature (i.e., T_g_). The spectrum of mechanical properties of the samples shown in Figure 2b confirms that the value of the T_g_ is higher than 180 °C. The highest peak in the mechanical spectrum, related to the glass transition, (i.e., α transition), is centered on 205 °C for the unfilled resin. The dispersion of an amount of filler of 2% slightly affects both storage modulus and tan δ. In particular, the highest peak is centered around 200 and 218 °C for 1D and 2D systems respectively. Moreover, there is a reduction in the modulus in the case of the MWCNT system (about 200 MPa in the range from −90 to 90 °C) and an increase of the same in the case of the EG composite (about 200 MPa in the range from −90 to 90 °C). Although the characteristics do not vary much with the inclusion of the filler, the configuration of the material structure has some substantial differences (see inset in Figure 2b). The glass transition can be influenced by both intramolecular and intermolecular interactions. Therefore, different factors imposed during the polymerization cycle, such as the incorporation of fillers and/or small molecules, affect the dynamic mechanical behavior. The height and width of the peaks in tan δ spectra provide additional information about the relaxation behavior. The profile of the tan δ, for epoxy resin in the temperature range from 120 to 280 °C, is composed of three superimposed peaks that suggest a wide distribution of relaxation mechanisms. This distribution is almost unchanged in the case of the 1D composite, while in the case of the 2D system a shoulder is observed before the main peak. This is shifted at a higher temperature with respect to the reference (epoxy) sample and the sample filled with MWCNTs. Compared to these, an enhancement of about 15 °C is observed. The significant change in the shape of tan δ for the 2D system suggests a different distribution of relaxation times than those observed for unfilled resin and for the samples filled with nanotubes. This behavior is most likely due to the extended interactions between rigid graphitic planes and polar groups of the resin network, which are limited only to the external wall in the case of MWCNTs of the 1D system. The formation of an intermediate layer of few nanometers thick on the resin-2D filler interface, as already reported in [33,34,35], is probably responsible for this marked enhancement. This different structural configuration may be one of the reasons why there is a greater electrical sensitivity to the mechanical deformation for the 2D system with respect to the MWCNT system, as described in the next section.

### 3.2. Piezoresistive Characterization

**Figure 3:**
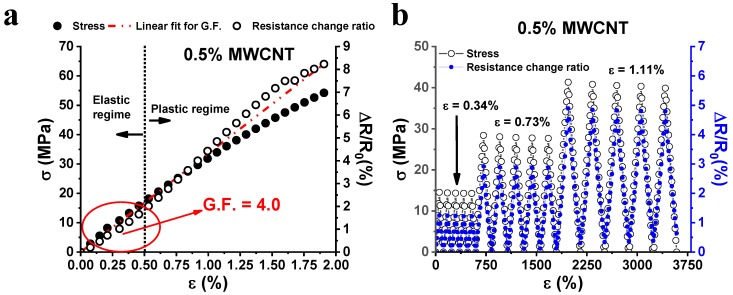
(**a**) Mechanical response (i.e., σ, left vertical axis) and resistance change ratio (i.e., ΔR/R0, right vertical axis) observed in tensile stress as function of the axial strain (ε); (**b**) Mechanical response (σ) and corresponding resistance change ratio (ΔR/R_0_) versus time under a progressively increasing maximum strain.

Figure 3a shows the electro-mechanical response of the sample with 0.5 wt.% of MWCNTs measured up to its failure (ε = 2%). The mechanical load plotted versus the axial strain is reported on the left vertical axis.

**Figure 4:**
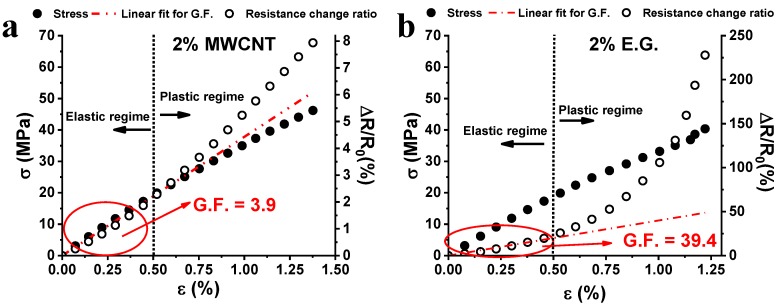
Mechanical response (i.e., σ, left vertical axis) and resistance change ratio (i.e., ΔR/R_0_, right vertical axis) observed in tensile stress as a function of the axial strain (ε) of: (**a**) MWCNTs composite at 2% by weight and (**b**) EG composite at 2% by weight.

The strain value of 0.5% discriminates the elastic/plastic regime of the material in which the mechanical response against the tensile stress is linear and nonlinear, respectively. In other terms, below this critical value, the material preserves its structural integrity whereas, beyond this value, it exhibits non-recoverable deformation (plastic regime) due to the irreversible modifications in the arrangement of the molecular segments in the resin network. Such a mechanism agrees with that concerning the resistance change ratio (right vertical axis, Figure 3a) caused by the applied axial tension. In fact, also with reference to this electrical property, in the first region corresponding to the elastic regime of the material, the value of ΔR/R_0_ shows an initial linear dependence with the applied strain. Then, when the strain threshold value of 0.5% is exceeded, a sharp change in the slope of the resistance change ratio is found. The irremediable deformations in the material due to mechanical stress cause the enlargement of the inter-tube distance and/or a decrease in the electrical contact areas. As discussed in [36], and analyzed in detail further on, this leads to an increase in the tunneling resistance between neighboring carbon nanotubes. From the slope of the line interpolating the experimental data of ΔR/R_0_ vs. strain, it is possible to compute the value of the gauge factor that is the usual parameter used to quantify the sensitivity of sensing materials. Figure 3b shows the results of cyclic tensile loading-unloading tests. A strain whose maximum value increases (i.e., 0.34%, 0.73%, and 1.11%) is applied and, synchronically, the resistance is detected. More in particular, when the strain falls in the elastic regime (i.e., ε = 0.34%), ΔR/R_0_ is null after each loading cycle because no significant permanent deformation has occurred in the sample. Instead, when strain falls in the plastic regime, (i.e., ε = 0.73% and ε = 1.11%) the presence of residual resistance is observed, after each loading cycle. For ε = 0.73%, the residual resistance found is small (i.e., ΔR/R_0_ ≈ 0.05%), because the strain value is still very close to the elastic/plastic regime threshold (ε = 0.5%). For a value of ε = 1.11%, the residual resistance found is higher (i.e., ΔR/R_0_ ≈ 0.3%), as the strain value lies in full plastic regime. Therefore, the microscale damages appear directly related to the resistance changes. Hence, they can be easily detected in a non-destructive way by detecting the residual resistance. This parameter increases with the increasing amount of plastic strain accumulated in the matrix. This behavior is due to the partial disconnections of the electrical network of MWCNTs embedded in the epoxy resin. As indicated before, if the plastic deformation induces an increase in the distance between neighbor CNTs aggregates larger around 2 nm or more, the tunneling effect becomes ineffective and thus the overall resistance increases. This effect is interesting because the sensor allows the detection of otherwise not viewable damages in the structure of the material [4,9]. Figure 4 shows the comparison of the piezoresistive properties of nanocomposites filled with 2 wt.% of MWCNTs and EG (see Figure 4a,b respectively).

Analogous considerations reported for the 0.5% MWCNTs system could be done for the system loaded with 2% MWCNTs. Except then for a slight decrease in elongation at break observed for the 2% MWCNTs system, the latter presents mechanical characteristics similar to those found for the MWCNTs 0.5% system. Also, the deformation threshold for which the system translates from an elastic regime to a plastic regime (ε = 0.5%) is similar. In addition, the trends of the change in the resistance ratio and the gauge factor (GF = 3.9) are similarly almost unchanged. The reason for this lies in the fact that the composites with 0.5% and 2% of MWCNTs show comparable electrical conductivity values (4.4 × 10^−2^ S/m and 6.1 × 10^−2^ S/m for the 0.5% and 2.0% MWCNTs systems, respectively). Therefore, there are no strong variations in the piezoresistive characteristics of the two samples, also because, in the considered concentration range, their conductivity lays in the high conduction region of the percolation curve (i.e., in the plateau region in Figure 1). In this regime, significant variations in electrical conductivity take place only due to notable increments of filler concentration. Although the composite with 2% of the expanded graphite shows similar mechanical characteristics to those exhibited by the MWCNTs-based system, it shows, in the same strain range, a very different resistance change ratio. In fact, for elongation of about 1%, the MWCNTs-based system achieves a ΔR/R_0_ value of about 4%, while the 2D filler -based system achieves a ΔR/R_0_ value of about 100%. The observed greater sensitivity to strain is confirmed by the high value of GF, equal to 39.4. To better understand the difference between 1D and 2D systems, it is necessary to discuss some aspects affecting the sensitivity of a system. In fact, processing conditions and material properties play a key role in determining the sensitivity value. Filler concentration, curing temperature, glass transition temperature and height of barrier values for the host matrix affect the tunneling resistance of the resulting nanocomposites [37]. As it concerns piezoresistive carbon nanotube-based systems, values of the gauge factor have been reported as ranging from 6 to 10 for matrices based on thermoplastic polymers [23,38,39] or epoxy resins characterized by low values of the glass transition temperature (71 °C) [39]. In the present study, a high GF was achieved using a structural epoxy resin characterized by high values in the storage modulus and a glass transition temperature higher than 180 °C (see Figure 2), which are fundamental requirements for its uses as structural material [5]. 

With regard to nanocomposites based on graphite derivatives, the GF of 39.4 observed in our case is much higher than other literature results, as reported in Table 2.

In other papers, the gauge factor has been evaluated in a non-linear strain range (GF = 45 or 65) [7,8] where the polymer is subjected to permanent deformations that make the material inapplicable for sensing scopes and dynamic stress cycles. In the case of the material here proposed, if the GF is calculated in the non-linear region of the curve ΔR/R_0_ vs. strain, its value ranges between 200 and 700 (the range is rather broad as expected for non-linear behavior). In other cases, epoxy resins are characterized by a GF of 56.7 [6]. However, such systems exhibit very low glass transition temperature (T_g_ of 130 °C) unacceptable for structural components.

**Figure 5:**
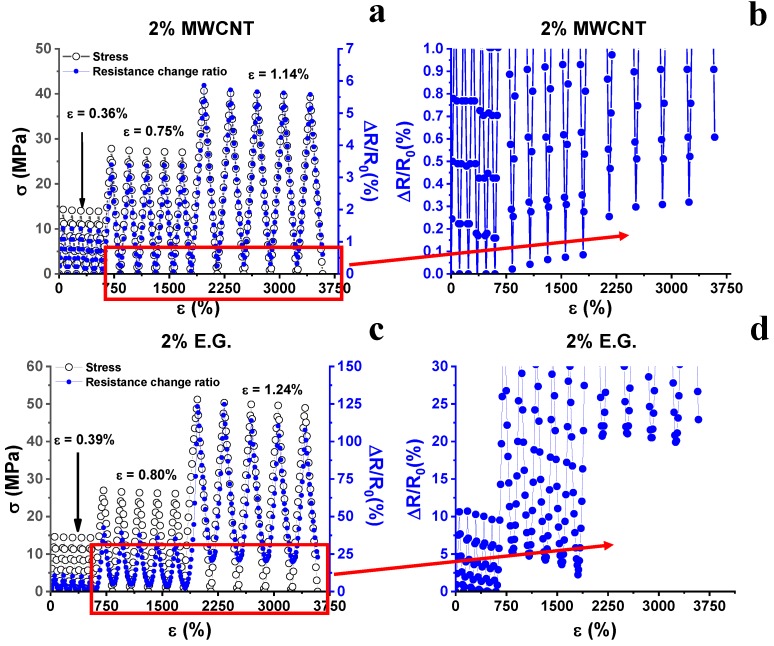
Mechanical response (σ) and corresponding resistance change ratio (ΔR/R_0_) versus time under a strain characterized by a progressively increasing maximum value: (**a**,**b**) MWCNTs composite at 2% by weight and (**c**,**d**) EG composite at 2% by weight.

As a consequence of the high sensitivity found for the 2D system, in the cyclic tensile loading–unloading tests up to a maximum strain of increasing value, the presence of the residual resistance is identified through ΔR/R_0_ values that are clearly higher than those obtained for the system 1D (see Figure 5). More specifically, bearing in mind that the maximum strains applied are comparable to those used for the 1D system, the same deformation (range between ε = 1.14 and ε = 1.24) is detected by a residual resistance of about 0.3% for the 1D system and 22% for the 2D system. The greater sensitivity of the 2D system allows evaluating with greater effectiveness also the deformations that are in the proximity of the value of the elastic/plastic threshold regime (ε = 0.5%).

### 3.3. Modeling of the Electro-Mechanical Mechanism

As discussed above, the piezoresistive properties of a strain sensor based on conductive filler-polymer nanocomposites were ascribed to the tunneling effect, which is the dominant mechanism for dc electrical conduction in such structures [44,45]. In particular, the overall resistance R of a nanocomposite can be calculated using the Equations (3) and (4), according to the model [1,46] derived from tunneling theory by Simmons [47]:

(3) $$R=\left(\frac{L}{N}\right)\left(\frac{8\pi \mathrm{hd}}{3{\gamma a}^{2}e^{2}}\right)\exp\left(\gamma d\right)$$

(4) $$\gamma =\frac{4\pi \sqrt{2{\mathrm{mE}}_{c}}}{h}$$

where L is the number of particles forming a single conducting path, N is the number of conductive paths, h is the Plank’s constant, d is the smallest distance between conductive particles, a^2^ is the effective cross-section area involved in the tunneling effect, e is the electron charge, m is the electron mass and E_c_ is the height of potential barrier between adjacent particles. During the axial tensile test, the resistance changes owing to particle separation and, more specifically, it increases due to the increase in interparticle distance. Assuming that the filler separation proportionally varies from d_o_ (the initial particle separation) to d, under the applied strain, the interparticle distance d can be expressed as follows [6,48]:

(5) $$d=d_{0}\left(1+k\varepsilon \right)$$

where k is a constant and ε is the strain elongation. Bearing in mind that for larger strain (plastic region) the resistivity increases faster, it is assumed that the number of conductive pathways changes at a much higher rate and can be expressed as follows [49]:

(6) $$N\left(\varepsilon \right)=\frac{N_{0}}{\exp\left(A\varepsilon +{B\varepsilon }^{2}+{C\varepsilon }^{3}+{D\varepsilon }^{4}\right)}$$

where N_0_ and N(ε) represent the initial amount of conducting paths and those at a specific deformation, respectively. A, B, C, and D are fitting parameters. Therefore, the resistance change ratio can be expressed using a combination of Equations (3)–(6) according to the following Equation:

(7) $$\frac{\Delta R}{R_{0}}=\frac{R-R_{0}}{R_{0}}=\frac{R}{R_{0}}-1=\left\{\left(1+k\varepsilon \right)\times \exp\left(A\varepsilon +{B\varepsilon }^{2}+{C\varepsilon }^{3}+{D\varepsilon }^{4}\right)\times \exp\left({\gamma d}_{0}k\varepsilon \right)\right\}-1$$

For ε values very close to zero, the exponential terms evolve toward the value 1 and therefore Equation (7) describes a linear relationship whose slope k represents the gauge factor (GF). In light of the above considerations, Equation (7) can be re-organized into the compact form:

(8) $$\frac{\Delta R}{R_{0}}=\left\{\left[1+\left(\mathrm{GF}\right)\varepsilon \right]\times {\left(\frac{N\left(\varepsilon \right)}{N_{0}}\right)}^{-1}\times \exp\left(\tau \varepsilon \right)\right\}-1$$

where

(9) $$\tau ={\gamma d}_{0}\left(G.F.\right)$$

**Figure 6:**
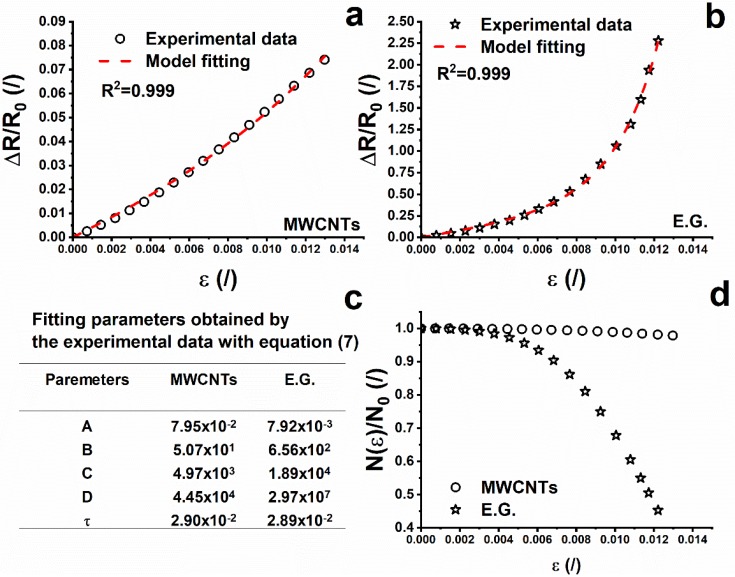
(**a**) Experimental data and theoretical predictions of resistance change ratio (ΔR/R_0_) as a function of strain for MWCNTs composite at 2% by weight and (**b**) EG composite at 2% by weight; (**c**) N(ε)/N_0_ as a function of strain of the systems at 2% by weight and (**d**) fitting parameters relating the Equation (7) of the systems at 2% by weight.

Figure 6a,b show the good accordance between experimental data and the behavior described by Equation (8); for both considered systems (R^2^ = 0.999). The fitting parameters are summarized in Figure 6c. 

From a careful evaluation of the fitting parameters, the term exp (τε) contributes to a minimum extent in Equation (8), as its value, for both systems, is approximately 1 in all the range of elongation. More specifically, the term exp (τε) differs from the value 1 for a maximum of 0.037% for the 1D system and of 0.035% for the 2D system evaluated at the maximum elongation value. In light of these considerations, Equation (8) can be approximated to the following Equation:

(10) $$\frac{\Delta R}{R_{0}}=\left\{\left[1+\left(\mathrm{GF}\right)\varepsilon \right]*{\left(\frac{N\left(\varepsilon \right)}{N_{0}}\right)}^{-1}\right\}-1$$

According to the tunneling effect, the electrical resistance between two adjacent conductive particles can be approximately estimated (Equation (2)) by Simmons’s theory. As mentioned in previous paragraphs, a critical distance between two particles of 2 nm has been identified. Due to the applied load, an increase in the strain leads to an increase in the tunneling distance between two adjacent conductive particles, which in turn induces a breakage of conductive pathways, thus leading to an increase in the electrical resistance. At the same time, some new conductive pathways are formed due to the reduction in tunneling distance among other particles not previously involved in conduction [48]. The breaking and formation of the conductive pathways coexist in the whole process. Since the breaking of the electrical paths predominates in the elongation process, an increase in ΔR/R_0_ is observed. Taking into account that the filler concentration, type of the resin and elongation during the tensile test (see Figure 4) are the same, the differences in the ΔR/R_0_ vs. strain can be ascribed to the filler dispersion, its aspect ratio, and the different interfacial contact area. The trend of ΔR/R_0_ vs. the strain is linear both for the CNT and EG systems in the elastic regime (less than ε = 0.5% in either case). In this region, where N(ε)/N_0_ is almost equal to 1 for both composites (see Figure 6d), the observed difference in the slope of the two systems, (i.e., GF) can only be associated to the parameter E_c_ of Equation (3) and d_o_ of Equation (5). It is important to point out that Equation (3) is valid for small deformations in correspondence of which growth of micro-crack does not occur. Knite et al. [50] introduced Equation (6) to consider non-reversible behavior related to the growth of micro-cracks which imposes an increase in the resistance higher than the one predicted by Equation (3). In other terms, Equation (6) describes a relation between the amount of conducting paths and the deformation, due to the fact that the destruction of conducting paths is an irreversible and cumulative phenomenon [6] in agreement with the results shown in Figure 6d. In fact, in the plastic regime, corresponding to permanent and irreversible deformations of the nanocomposite structure, the ratio N(ε)/N_0_ slightly decreases with the strain in the case of the CNT system and more significantly in the case of the EG system. Given the key role of the aspect ratio and interfacial contact area in determining the piezoresistive behavior of the nanostructured resins, morphological analysis was carried out in order to investigate the possible impact of such influencing parameters.

### 3.4. Morphological Analysis

**Figure 7:**
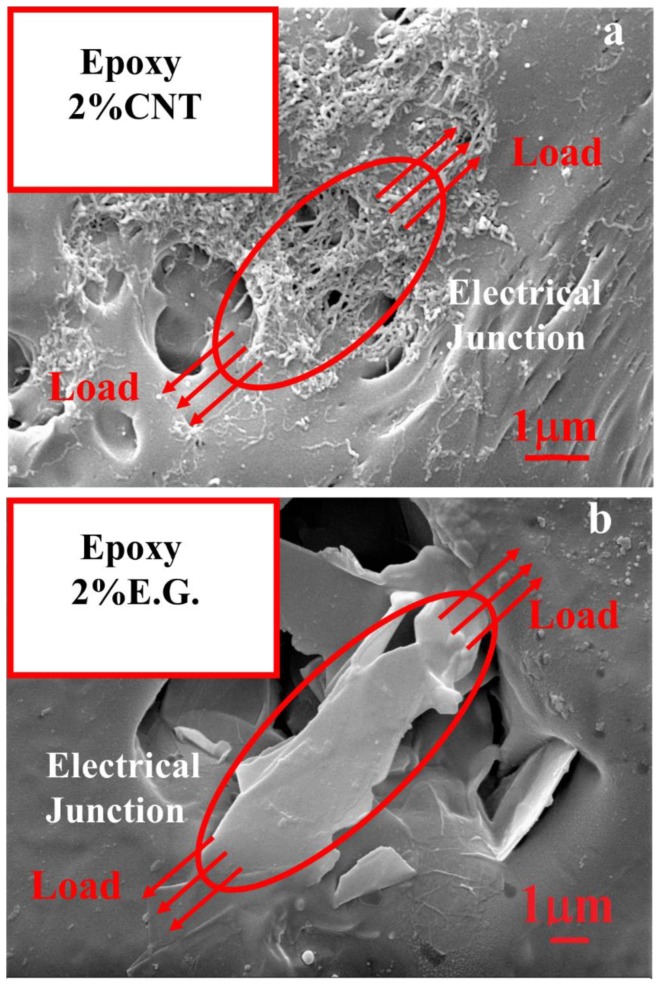
Scanning electron microscopy (SEM) images of the fracture surface of (**a**) MWCNTs composite at 2% by weight and (**b**) EG composite at 2% by weight.

Figure 7 shows the SEM images of the composites filled with 2% of MWCNT (Figure 7a) and 2% of EG (Figure 7b) where an etching reactant was employed to partially remove the epoxy resin to explore more accurately the arrangement of the fillers within the matrix. 

Carbon nanotubes appear as a densely interconnected network like a series of tangles forming a compact conductive surface. During the mechanical test, the applied load determines the breaking of the pathways formed by the tangled carbon nanotubes leading to an increase in the electrical resistance. The piezoresistive response is not relevant both for the formation of new conductive networks by connecting neighboring nanotubes [51] and also because the series of tangles of carbon nanotubes appear to be difficult to disentangle, as shown in the SEM images, and in agreement with the trend of the ratio N(ε)/N_0_ vs. the strain, reported in Figure 6d. N(ε)/N_0_ differs slightly from the value 1 in the entire investigated strain range. This indicates that the number of conductive paths is only slightly reduced. Otherwise, for the 2D system, N(ε)/N_0_ deviates from the unit value up to the value of 0.45, most likely due to a strong reduction in the number of conductive pathways. The differences in the aspect ratio and in the particle contact area of the two fillers differently affect the arrangement of the percolating network under load.

For EG-based composites, given the tight arrangement of the layers, the application of a load leads to a sliding phenomenon between conductive layers. As a result, an increase in the interlayer distances and the reduction in the overlapping area among particles involved in the electron tunneling is observed, which enhances the overall resistivity of the composite. In light of these considerations, the higher sensitivity behaviour against mechanical strain for the 2D system compared to that of the 1D system [51] can be explained. In fact, the larger inter-contact area among the graphite layers, due to their 2-D structure, may cause a larger contact resistance change than that occurring for a 1-D structure such as the carbon nanotubes [32,52].

**Figure 8:**
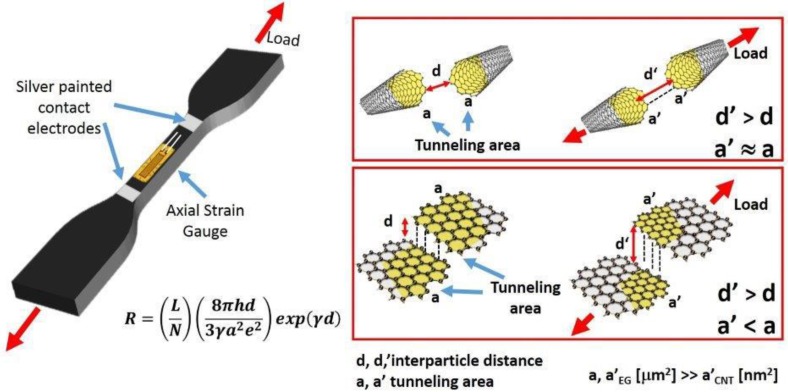
Scheme of involved areas in the tunneling phenomenon for 1D and 2D system.

Moreover, the SEM images also reveal an evident difference in the electrical junctions (EJ) formed in the resin (see red circles) which is larger in the case of the 2D system compared to those established by dispersing MWCNTs. This confirms once again the double contribution (interparticle distance and their effective areas involved in the tunneling phenomenon) in determining the best piezoresistive response of graphene-based composites with respect to those based on nanotubes. In this latter case, the tunneling area is limited to sections of the order of few nanometers (see Figure 8). 

Further investigation is underway to investigate if the greater rigidity (storage modulus and tan δ) of 2D systems may also affect the height of barrier E_C_ of Equation (3). In fact, such a parameter is widely accepted as one strongly conditioning the electrical response of nanocomposites [29,53].

## 4. Conclusions

The sensing properties of a structural nanocomposite material obtained from epoxy resin with a high glass transition temperature suitable for structural applications have been investigated. Two different carbon-based fillers, multi-wall carbon nano tubes and exfoliated graphite characterized respectively by 1D and 2D structures and different aspect ratios, have been incorporated in the same insulating resin. The piezoresistive response of the resulting nanocomposites evidences different values of the gauge factor. Values of four and 39 have been found for composites filled with 1D and 2D particles, respectively. The greater sensitivity of the 2D system against permanent deformations has been interpreted in light of morphological considerations and an analytical model. The results highlight the relevant role of the filler aspect ratio on the GF of the developed nanocomposites. In particular, the difference in the aspect ratio of the two different fillers strongly influences the particle contact area, affecting the arrangement of the percolating network under load. The difference in the change in the contact resistance among graphite layers and CNTs dispersed in the resin seems to be relevant in determining the sensitivity of the formulated nanocomposites.

The proposed nanocomposites appear as promising candidates for applications, such as in the aeronautic sector, where structural health monitoring assumes particular relevance for safety issues.

## Figures and Tables

**Table 1:** DMA technical specifications.

Operating Conditions	Specific
Sample dimension	1 × 10 × 25 mm^3^ (thickness, width, and length, respectively)
Configuration	3-points bending mode
Displacement amplitude	0.02 mm
Frequency operating condition	1 Hz
Temperature operating condition	from −90 °C to 280 °C
Scanning rate	3 °C/min^−1^

**Table 2:** Gauge factor for different graphene-based composites.

Nanocomposite	Gauge Factor	Ref.
Graphene-CNT/epoxy composites	10	[16]
Graphene ribbons	1.9	[40]
Graphene/PDMS	6.1	[41]
Graphene/TPU	5.2	[42]
Graphene nanocellulose/PDMS	7.1	[43]
Epoxy-Expanded Graphite	39.4	[This paper]
